# Case Report: Severe Hypercalcemia Following Vitamin D Intoxication in an Infant, the Underestimated Danger of Dietary Supplements

**DOI:** 10.3389/fped.2022.816965

**Published:** 2022-02-01

**Authors:** Alexandre O. Gérard, Audrey Fresse, Myriam Gast, Diane Merino, Pierre Gourdan, Audrey Laurain, Irène Margaritis, Pierre-Alexis Gauci, Fanny Huret, Nadège Parassol, Fanny Rocher

**Affiliations:** ^1^Department of Nephrology-Dialysis-Transplantation, Centre Hospitalier Universitaire de Nice, Nice, France; ^2^Department of Pharmacology and Pharmacovigilance Center of Nice, Centre Hospitalier Universitaire de Nice, Nice, France; ^3^Department of Pediatry, Hospital Center of Bastia, Bastia, France; ^4^Department of Pediatry, Lenval Hospital - Centre Hospitalier Universitaire de Nice, Nice, France; ^5^Nutrition Risk Assessment Unit, French Agency for Food, Environmental and Occupational Health & Safety (ANSES), Maisons-Alfort, France; ^6^Department of Gynecology, Centre Hospitalier Universitaire de Nice, Nice, France

**Keywords:** vitamin D, overdose, intoxication, dietary supplement, hypercalcemia, pharmacovigilance, misuse, case report

## Abstract

Vitamin D supplementation is routinely introduced in infants, according to medical guidelines. However, vitamin D overdose can result in life-threatening hypercalcemia. We report the case of a 3-month-old infant who suffered from severe hypercalcemia. Upon detailed questioning of the parents, a vitamin D administration error has been identified. Indeed, the parents had followed the advice of their midwife. They substituted the prescribed medicinal vitamin D by a dietary supplement, different in concentration and dosing, without performing the dose conversion needed. In fact, many different medications and dietary supplements with vitamin D exist, offering various concentrations and units of measurement. This case highlights the pivotal role of therapeutic education. Broadly, there is a need for harmonization of the regulation and labeling of dietary supplements and medications containing vitamin D.

## Introduction

Vitamin D is a fat-soluble hormone either synthetized endogenously or from external vitamin intakes. Vitamin D bears pleiotropic functions (1, 2), primarily on phosphocalcic and bone homeostasis, crucial in infants (3). The French Agency for Food, Environmental and Occupational Health & Safety (ANSES) (4) and the European Food Safety Authority (EFSA) have revised the nutritional guidance for vitamin D. The considered Adequate Intake (AI) for infants <6 months is 10 μg or 400 IU per day. In this context, according to guidelines recommendations, vitamin D supplementation is routinely initiated in infants (5) under medical control. The Institute of Medicine (IOM) and the EFSA have also defined Upper tolerable Levels (UL) of vitamin D according to the age (6, 7), even if the threshold for acute toxicity is still unclear (3). Based on the risk of growth perturbation and hypercalcemia, the vitamin D UL for children <6-months, is 25 μg or 1,000 IU per day (4, 7, 8). We report a case of vitamin D overdose in an infant, caused by the erroneous substitution of the prescribed supplementation by a dietary supplement.

## Case Description

In August 2020, a 3-month-old infant was referred to the pediatric emergency department for severe anorexia, vomiting, and weight loss. After an uncomplicated pregnancy (36 weeks and 5 days of amenorrhea), he was born at term, weighing 2.3 kg. Since, he had been exclusively breastfed and had not presented any medical problem.

At the time of admission, the infant weighted 4.5 kg. He had no fever, his heart rate was 109 beats per minute, his blood pressure was 107/85 mmHg, and his oxygen saturation was 99%. Blood pressure was later reassessed several times, and was found normal or at the upper limit of normal for the age. Clinical examination was unremarkable except a global hypotonia and the presence of moderate dark circles around the eyes. Natremia was 139 mmol/L, kalemia 4.4 mmol/L, alkaline reserve 21 mmol/L, hemoglobin 9.1 g/dL, leukocytes 11.11 G/L, platelets 471 G/L, and C-reactive protein (CRP) 13 mg/L with a negative procalcitonin. Blood gases analysis and liver function tests were within normal range. Natriuresis was <20 mmol/L for a kaliuresis of 33 mmol/L, suggestive of hypovolemia. Hemoculture, stool bacterial culture and virology, as well as lumbar puncture results were all negative. Lumbar puncture had been performed because the infant presented with global hypotonia and severe behavioral changes, in the absence of an obvious diagnosis. Immunoglobulin E (IgE) antibodies to cow milk were negative. Cranial ultrasonography and brain Magnetic Resonance Imaging (MRI) were normal. Five days after admission, an abdominal ultrasound confirmed 3 days later revealed some deposits in the bladder, which were deemed clinically not significant.

Because of a suspicion of urinary infection, probabilistic intravenous antibiotic therapy was initiated, then discontinued 3 days later as no inflammatory syndrome nor bacteria in urinary analysis (including mycobacteria in a context of sterile leukocyturia) were found. Breastfeeding was supplemented with infant milk and intravenous hydration.

Calcemia was assessed for the first time 12 days after admission to hospital and was at 3.08 mmol/L (normal values: 2.15–2.55 mmol/L) with an albuminemia of 41 g/L (normal values: 34–42 mg/L). Parathormone (PTH) was below the limit of quantification (normal values: 18–88 ng/L), whereas calcidiol was above the upper limit of quantification (normal values: 30–400 ng/mL) and calcitriol was 200 pg/mL (normal value <182 pg/mL). Phosphate was 1.8 mmol/L (normal values: 1.6–2.4 mmol/L). Serum creatinine was 23 μmol/L (normal values: 15–37 μmol/L) while urea was 2.3 mmol/L (normal values: 1.8–6.4 mmol/L). Urinary calcium was 2.75 mmol/L with a creatininuria of 1 mmol/L.

Electrocardiogram was normal and no arrhythmia was documented on the monitoring. The infant was kept hydrated and calcemia progressively decreased to reach 2.78 mmol/L in mid-September. Bisphosphonates were not deemed necessary. The child was finally discharged with a weight of 4.8 kg and with a close monitoring of his calcemia recommended. Throughout a follow-up consultation, 2 weeks later, calcemia was 2.6 mmol/L and the child weighed 5.2 kg. On last follow-up consultation 3 months after the onset of symptoms, he weighed 5.6 kg and was clinically asymptomatic. Renal ultrasound was suggestive of calcic deposits in the urinary tract.

Upon detailed questioning of the parents, a chronic overdose of vitamin D was identified. In order to provide the recommended daily dose for breastfed newborn, the maternity medical staff initially had prescribed 4–5 drops per day of ZymaD®, a brand name containing cholecalciferol dosed at 10,000 International Unit (IU)/mL, one drop containing 300 IU of vitamin D (9). Once at home, a caring midwife suggested to the parents to replace the initially prescribed vitamin D supplementation by a so-called “natural” vitamin D-based dietary supplement (DS). She indeed considered that “classical” drugs including vitamin D may contain endocrine disrupters, as well as preservatives, and believed that “natural” vitamin D, marketed as DS should be healthier. She was also convinced that DS would be associated with less abdominal pain in newborn. The midwife suggested several DS, available on the Internet, and let the parents pick one of them. She recommended the parents to maintain “the same dose” as the one initially prescribed (for ZymaD®).

Thereupon, the parents bought on the Internet the DS Sunday Natural® brand of D3 10,000 IU (+ vitamin K2). This product is deemed to contain 10,000 IU *per* drop (and not *per* mL), and the manufacturer's recommendation consists of one drop every 10 days. However, as initially prescribed with ZymaD®, 4–5 drops per day were administered. This switch between two non-equivalent drugs, with different dosing, consequently exposed the infant to 40,000–50,000 IU per day, which represents 50-fold the Upper tolerable Level (UL) recommended. Such exposition conducted to a symptomatic severe vitamin D overdose, with a 15-day hospital stay and potential sequelae such as nephrocalcinosis and lithiasis.

## Discussion

This case highlights the pivotal role of health practitioners in limiting the risk of drug and dietary supplement misuse, even for so-called innocuous “vitamins.” Because of different regulatory specific domains, blurred frontiers between recommended use and potentially harmful errors may lead to serious health hazards. This situation may become more and more frequent, especially considering the current hype surrounding vitamin D.

Vitamin D overdose is suspected when hypercalcemia coexists with calcidiol > 150 ng/mL (10) and low plasmatic levels of parathormone (11, 12). The clinical manifestations result from the subsequent acute hypercalcemia: confusion, polyuro-polydipsia and dehydration, anorexia, various transit troubles, cardiac rhythm and conduction disorders. In this case, the infant likely presented severe symptomatic hypercalcemia, as it was > 3 mmol/L despite hydration, the 12th day of hospitalization. Indeed, he probably suffered from a delay in diagnosis, as hypercalcemia was not suspected until the 2nd week of hospitalization. Early assessment of calcemia would have dramatically changed the management of this patient, preventing diagnostic delay and unwarranted investigations. Acute intoxication with calcitriol lasts a few days only, because of its short half-life. Conversely, calcidiol has a high affinity for its transport binding protein, hence a circulating half-life of 2–3 weeks (13), and accumulates in adipose tissue (14) and liver (15). Therefore, intoxication with calcidiol can last for months.

Vitamin D intoxications have been reported in patients consuming large doses of vitamin D-containing supplements, either voluntarily or as medication and dietary supplement errors. The latter pertain to the multiple brands, types of packaging and formulations of dietary supplements, resulting in prescription or administration errors (11, 13, 16, 17).

In this case, the lack of communication and/or comprehension between the parents and the midwife resulted in an error in drug administration. It took roots in substituting the medicinal vitamin D by a dietary supplement of different dosing. The risk is further increased by the plethora of alternate formulas available on the Internet, with various concentrations and expression of dosages. Up to 28 pharmaceutical brands of vitamin D are available in France, not to mention the DS and the various multivitamin complexes. The labeling of vitamin D products (drugs and dietary supplements) suffers from a lack of harmonization, misleading the consumer and exposing to hazardous titrations.

Besides, products containing vitamin D are framed by two distinct regulations, regarding their qualification as drug or dietary supplement. Indeed, the sole notification to the competent authorities without any objection within 2 months allows the manufacturer to put a dietary supplement on the market. Manufacturers are free to mislead the consumer to benefit from the aura of the purported naturalness of their DS, maintaining confusion with equivalent drugs. However, vitamin D content of unlicensed DS is believed to vary widely from labeled claims, as DS are manufactured under less stringent quality standards (18). Yet, patients consuming DS are seldom suspicious toward those products, considered innocuous and branded as healthy, while they consider drugs with Marketing Authorization Holders as dubious. Indeed, the increasing use of DS is partly due to the presence in drugs of antioxidant excipients such as butylhydroxytoluene (BHT), considered harmful by parents and by some health professionals. Anyhow, some drugs are exempt of BHT, but are not reimbursed in most cases (e.g., Deltius®).

The error risk when dispensing or administering medications is further increased during the postpartum period, upon returning home. Mother and child both have their own prescriptions, sometimes including the same pharmacological classes or presentations (e.g., vitamin D, vaccines) exposing to potential errors. Parents are receptive to marketing arguments insisting on the naturalness of the products intended for their child.

Our case highlights the risk of vitamin D overdose, particularly in the pediatric population. The COVID-19 pandemic sparked interest regarding the potential protective effect of vitamin D and there is elsewhere growing hype regarding its potential role in cancer or immunity *inter alia* (12). This may lead more and more patients to the temptation of automedication and/or misuse of products containing vitamin D. Indeed, more than three quarters of reports about vitamin D intoxication have been published from 2010 (17). In this setting conducive to mistakes, actions should be taken to minimize as much as possible all add-on preventable sources of errors, insomuch as most cases of vitamin D intoxication could be easily preventable (17).

Whatever the drug, there is a need for patients to be adequately informed and instructed on how to administrate the appropriate dosage. Anyhow, keeping in mind the risk of vitamin D intoxication, early assessment of calcemia is pivotal to the diagnostic approach of unexplained behavioral change and anorexia in infants. The present report and two others fostered ANSES to issue a warning to recommend drugs containing vitamin D over DS (19, 20). Broadly, there is a real need for harmonization of the regulation, dosing and recommendations for use of dietary supplements.

## Data Availability Statement

The original contributions presented in the study are included in the article/Supplementary Material, further inquiries can be directed to the corresponding author/s.

## Ethics Statement

Written informed consent was obtained from the minor(s)' legal guardian/next of kin for the publication of any potentially identifiable images or data included in this article.

## Author Contributions

All authors listed have made a substantial, direct, and intellectual contribution to the work and approved it for publication.

## Conflict of Interest

The authors declare that the research was conducted in the absence of any commercial or financial relationships that could be construed as a potential conflict of interest.

## Publisher's Note

All claims expressed in this article are solely those of the authors and do not necessarily represent those of their affiliated organizations, or those of the publisher, the editors and the reviewers. Any product that may be evaluated in this article, or claim that may be made by its manufacturer, is not guaranteed or endorsed by the publisher.
